# Supplementary material for: Evidence-based teaching practices correlate with increased exam performance in biology
Source: PLoS One. 2021 Nov 30;16(11):e0260789. doi: 10.1371/journal.pone.0260789 (PMC8631643; doi:10.1371/journal.pone.0260789)
Supplement: S1 Table — (PDF) [file pone.0260789.s001.pdf]

	Female	Male	Total
**Lecturer**	12	4	16
**Tenure Track**	8	9	17
**Total**	20	13	33
