# Supplementary material for: Evidence-based teaching practices correlate with increased exam performance in biology
Source: PLoS One. 2021 Nov 30;16(11):e0260789. doi: 10.1371/journal.pone.0260789 (PMC8631643; doi:10.1371/journal.pone.0260789)
Supplement: S2 Table — (PDF) [file pone.0260789.s002.pdf]

**Teaching Type**	**Number of Coded Videos**		**Total**
	Three	Four	
Team-Taught	6	10	16
Solo-Taught	28	2	30
**Total**	34	12	46
