# Supplementary material for: Evidence-based teaching practices correlate with increased exam performance in biology
Source: PLoS One. 2021 Nov 30;16(11):e0260789. doi: 10.1371/journal.pone.0260789 (PMC8631643; doi:10.1371/journal.pone.0260789)
Supplement: S3 Table — (PDF) [file pone.0260789.s003.pdf]

Teaching Type	Number of Coded Videos		Total
	Three	Four	
Team-Taught	6	8	14
Solo-Taught	27	1	28
**Total**	33	9	42
