# Supplementary material for: Evidence-based teaching practices correlate with increased exam performance in biology
Source: PLoS One. 2021 Nov 30;16(11):e0260789. doi: 10.1371/journal.pone.0260789 (PMC8631643; doi:10.1371/journal.pone.0260789)
Supplement: S4 Table — BIC = Bayesian Information Criterion; ABIC = Adjusted BIC; BLRT = Bootstrap Likelihood Ratio Test; LMRT = Lo-Mendell-Rubin Adjusted Likelihood Ratio Test.; BF = Bayes Factor; cmP = Correct Model Probability; SIC = Schwarz Information Criterion. Best fit statistics are in boldface. (PDF) [file pone.0260789.s004.pdf]

Number of Profiles	Log likelihood	BIC	ABIC	p-value of BLRT	p-value of LMRT	Entropy	BF	cmP
1	-6270.49	12682.74	12594.10	-	-	-	7.5E-166	3.5E-231
2	-5852.31	**11922.31**	11786.19	< .001	**0.0003**	0.981	1.1E-40	4.7E-66
3	-5722.36	11738.35	11554.75	< .001	0.0918	0.987	1.9E-10	4.2E-26
4	-5662.01	11693.58	11462.5	< .001	0.7783	0.975	2.0E-09	2.2E-16
5	-5603.99	11653.49	11374.93	< .001	0.3477	0.975	1.1E-07	1.1E-07
6	**-5550.01**	11621.47	11295.43	< .001	0.6573	0.974	0	1
