# Supplementary material for: Evidence-based teaching practices correlate with increased exam performance in biology
Source: PLoS One. 2021 Nov 30;16(11):e0260789. doi: 10.1371/journal.pone.0260789 (PMC8631643; doi:10.1371/journal.pone.0260789)
Supplement: S5 Table — *p < 0.05, **p < 0.01, ***p < 0.001. (PDF) [file pone.0260789.s005.pdf]

Practice	1	2	3	4	5	6	7	8	9	10	11	12	13	14
**1. HB**	-													
**2. Alone**	0.60 **	-												
**3. SG**	0.71 **	0.40 **	-											
**4. Exp_Ans**	0.57 **	0.17	0.73 **	-										
**5. Alt_Ans**	-0.01	0.17	-0.02	-0.15	-									
**6. ST_DB**	0.63 **	0.29	0.60 **	0.73 **	-0.19	-								
**7. TST**	0.59 **	0.40 **	0.71 **	0.68 **	0.01	0.82 **	-							
**8. DB**	0.58 **	0.14	0.72 **	0.89 **	-0.15	0.80 **	0.71 **	-						
**9. Vol_Ans**	0.19	-0.13	0.18	0.28	-0.15	0.21	0.09	0.31 *	-					
**10. RC_Ans**	0.66 **	0.24	0.77 **	0.83 **	-0.21	0.82 **	0.74 **	0.89 **	0.21	-				
**11. Ins_Exp**	0.29	0	0.25	0.09	-0.1	0.09	0.07	0.25	0.41 **	0.16	-			
**12. Vol_Exp**	0.49 **	0.17	0.50 **	0.84 **	-0.13	0.57 **	0.47 **	0.72 **	0.29	0.52 **	0.02	-		
**13. PFBS**	0.75 **	0.42 **	0.71 **	0.74 **	0.04	0.80 **	0.74 **	0.71 **	0.21	0.79 **	0.23	0.50 **	-	
**14. PK**	0.56 **	0.27	0.59 **	0.38 *	0.14	0.27	0.31 *	0.48 **	0.29	0.57 **	0.26	0.24	0.35 *	-
