# Supplementary material for: Evidence-based teaching practices correlate with increased exam performance in biology
Source: PLoS One. 2021 Nov 30;16(11):e0260789. doi: 10.1371/journal.pone.0260789 (PMC8631643; doi:10.1371/journal.pone.0260789)
Supplement: S6 Table — Mean values with standard deviation in parentheses. n all units = 46, n high Bloom’s = 24, n low Bloom’s = 22. (PDF) [file pone.0260789.s006.pdf]

**Practice**	**All Exams**	**High Bloom's Exams**	**Low Bloom's Exams**
HB	2.27 (2.67)	3.10 (2.94)	1.36 (2.04)
MCQ	0.87 (1.33)	1.07 (1.56)	0.66 (1.01)
SA	2.42 (4.42)	2.96 (4.27)	1.84 (4.62)
Alone	1.52 (1.60)	1.84 (1.72)	1.18 (1.42)
SG	4.29 (4.88)	4.95 (4.40)	3.57 (5.35)
DB	8.72 (7.50)	10.10 (8.38)	7.21 (6.25)
Ins_Ans	6.20 (4.65)	5.91 (3.95)	6.52 (5.38)
Vol_Ans	6.63 (6.43)	5.96 (3.96)	7.36 (8.39)
RC_Ans	2.36 (4.41)	3.27 (4.93)	1.37 (3.63)
WC_Ans	1.76 (3.67)	2.03 (4.39)	1.47 (2.77)
Ins_Exp	7.87 (4.59)	7.94 (3.57)	7.80 (5.58)
Vol_Exp	1.96 (2.30)	2.55 (2.81)	1.31 (1.33)
RC_Exp	1.17 (2.58)	1.40 (2.44)	0.93 (2.77)
Exp_Ans	3.12 (3.95)	3.93 (4.36)	2.24 (3.33)
STDB	2.80 (4.22)	3.71 (5.03)	1.81 (2.91)
Prom_Log	1.14 (1.75)	1.45 (1.82)	0.80 (1.65)
Alt_Ans	1.08 (1.27)	0.92 (1.08)	1.25 (1.45)
PFBS	2.05 (2.65)	2.78 (2.89)	1.25 (2.15)
PFBC	1.40 (1.52)	1.66 (1.50)	1.11 (1.51)
PK	0.50 (0.54)	0.56 (0.55)	0.44 (0.53)
TST	10.01 (8.52)	12.58 (8.51)	7.20 (7.77)
