# Supplementary material for: Evidence-based teaching practices correlate with increased exam performance in biology
Source: PLoS One. 2021 Nov 30;16(11):e0260789. doi: 10.1371/journal.pone.0260789 (PMC8631643; doi:10.1371/journal.pone.0260789)
Supplement: S1 Fig — (PDF) [file pone.0260789.s007.pdf]

Observer ___________ Date of Observation: ______________________ Page ___ of _____

School: _______________ Course: ______________ Instructor: ________________ Session Date: ___________

1. Start of Class (Min:Sec): _______________ 2. End of Class (Min:Sec): _____________

	Observations:		Activity ___	Activity ___	Activity ___	Activity ___	Activity ___
Intro	3. Bloom Level of Activity:		H L	H L	H L	H L	H L
	4. Form of Activity/Question:		MCQ W One SA	MCQ W One SA	MCQ W One SA	MCQ W One SA	MCQ W One SA
SE: Iteration 1	5. Start (min:sec):						
	6. End (min:sec):						
	7. Question discussed (Type of SE):		I SG	I SG	I SG	I SG	I SG
	8. Voting included: Yes (Y), No (N)		Y N	Y N	Y N	Y N	Y N
	9. Is the correct answer in anyway indicated?		Y N	Y N	Y N	Y N	Y N
SE: Iteration 2	10. Start (min:sec):						
	11. End (min:sec):						
	12. Question discussed (Type of SE):		I SG	I SG	I SG	I SG	I SG
	13. Voting included: Yes (Y), No (N)		Y N	Y N	Y N	Y N	Y N
	14. Is the correct answer in anyway indicated?		Y N	Y N	Y N	Y N	Y N
SE: Iteration 3	15. Start (min:sec):						
	16. End (min:sec):						
	17. Question discussed (Type of SE):		I SG	I SG	I SG	I SG	I SG
	18. Voting included: Yes (Y), No (N)		Y N	Y N	Y N	Y N	Y N
	19. Is the correct answer in anyway indicated?		Y N	Y N	Y N	Y N	Y N
Debrief	20. Start of Debrief (min:sec):						
	21. End of Debrief (min:sec):						
	22. Who gives the answer?	Instructor (Y/N)					
		# of Volunteers					
		# of Random Calls					
		# of Whole Class					
	23. Who gives the explanation?	Instructor (Y/N)					
		# of Volunteers					
		# of Random Calls					
		No Explanation (N)					
	24. Length of instructor time for teaching/answering/explaining during debrief (min:sec):						
Activity Characteristics: Can occur at any time	25. Focus on **logic** (# of times):						
	26. Number of **alternative (or wrong) answers explained**:						
	27. How many times students **asked Qs** to the WC **during activity**?						
	28. **Positive feedback** (# of times):	Entire Class (C)					
		Individual Student (S)					
	29. **Negative feedback** (# of times): C/S						
	30. **Prior knowledge** (# of times):						
	31. Praise **effort/improvement** over ability (# of times):						
	32. **Errors are natural and useful/educational** (# of times):						
	33. How many **Qs aside from activity**?						
	34. Comments:						
