# Supplementary figures and images for: Evidence-based teaching practices correlate with increased exam performance in biology
Source: PLoS One. 2021 Nov 30;16(11):e0260789. doi: 10.1371/journal.pone.0260789 (PMC8631643; doi:10.1371/journal.pone.0260789)

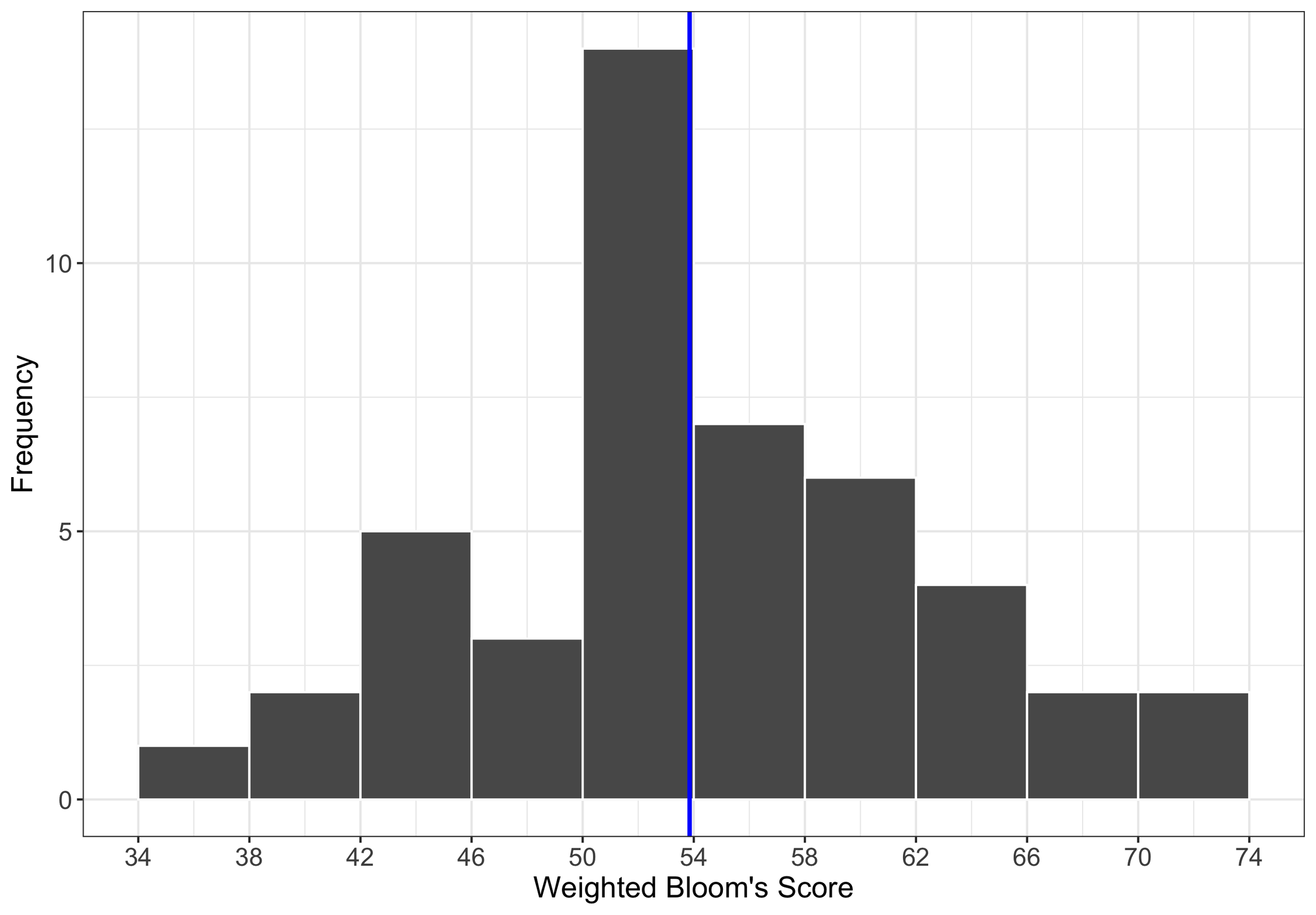

Supplement: S2 Fig — Distribution of the weighted Bloom’s scores of biology exams for all 46 units of analysis. BL = Weighted Bloom’s level of exams. Median = 53.8, SD = 8.00. Median value is shown as a blue vertical line. (TIF) [file pone.0260789.s008.tif]

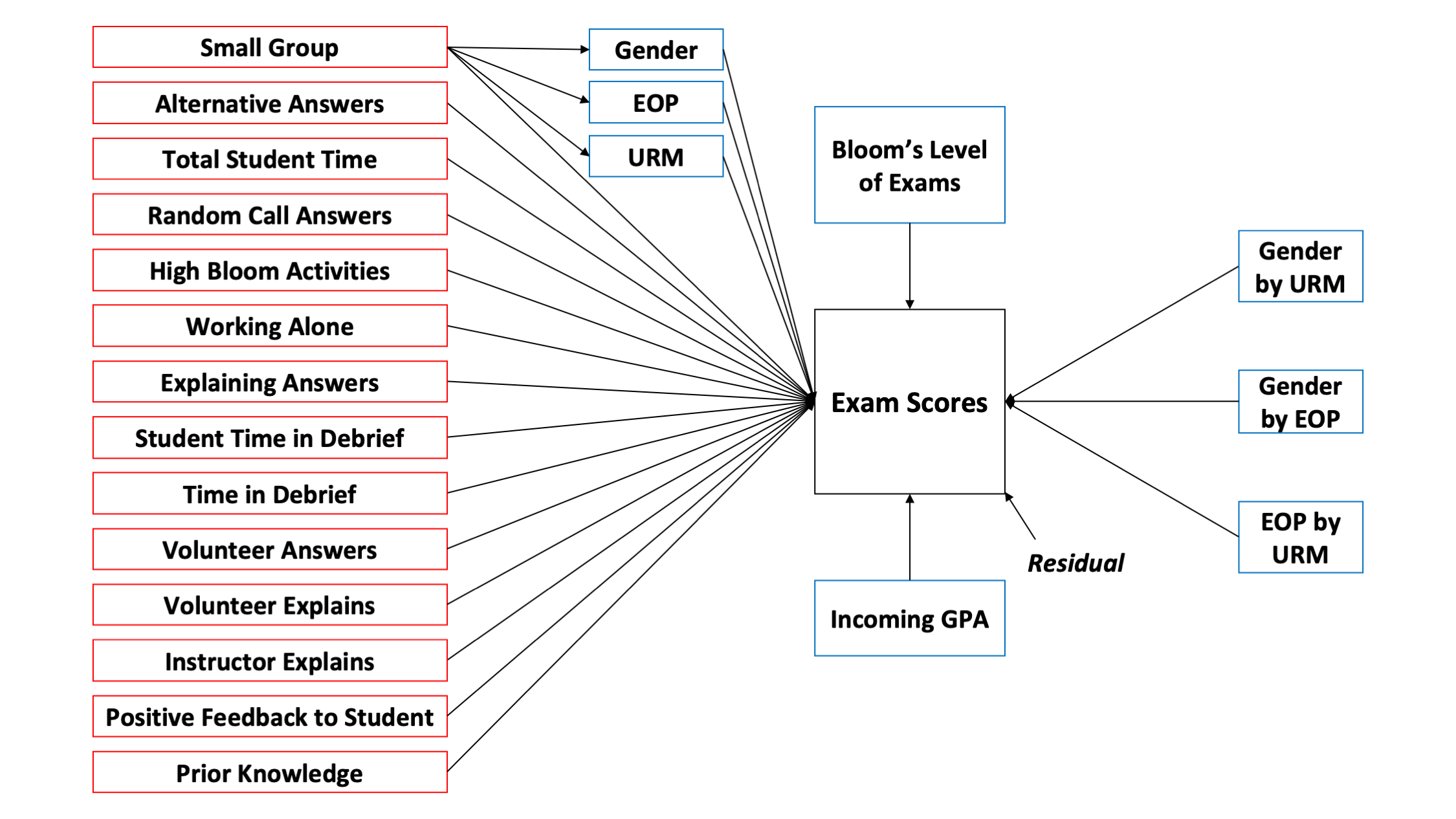

Supplement: S3 Fig — All 14 PORTAAL practices with demographic variables as mediators were tested but are only shown for small group activities to reduce complexity of the figure. PORTAAL practice boxes are in red. Covariates, interactions between demographic variables, and mediator boxes are in blue. (TIF) [file pone.0260789.s009.tif]
