# Supplementary material for: Evidence-based teaching practices correlate with increased exam performance in biology
Source: PLoS One. 2021 Nov 30;16(11):e0260789. doi: 10.1371/journal.pone.0260789 (PMC8631643; doi:10.1371/journal.pone.0260789)
Supplement: Attachment — Submitted filename: Response to Reviewers.pdf [file pone.0260789.s012.pdf]

DEPARTMENT OF BIOLOGY
UNIVERSITY of WASHINGTON

September 15, 2021

To: Dr. Paulo F. Carvalho, Academic Editor PLOS ONE
From: Dr. Mary Pat Wenderoth
Concerning: Manuscript PONE-D-21-24236
Evidence-based teaching practices correlate with increased exam performance in biology

Dear Dr. Paulo F. Carvalho,

Please thank the reviewers for their very helpful comments on our manuscript. By addressing the issues that they raised, we feel that we have made this a stronger paper that will be of great interest to the PLOS ONE readership.

We are submitting the three documents you requested.

- A rebuttal letter that responds to each point raised by the academic editor and reviewer(s) uploaded as a separate file labeled 'Response to Reviewers'.
- A marked-up copy of your manuscript that highlights changes made to the original version. This is uploaded as a separate file labeled 'Revised Manuscript with Track Changes'.
- An unmarked version of your revised paper without tracked changes. This is uploaded as a separate file labeled 'Manuscript'.

Below are our responses to each of the editor or reviewers' comments. Our responses are in blue italicized text immediately following the comment.

RESPONSES

Finally, in your Data Availability statement you state that all data is available without restriction. However, I did not see any link to a data repository and the data provided in the Supplementary Materials seems to be only group averaged results or statistical tables. To comply with PLOS ONE Data Availability policy, I encourage you to make the raw coding data available through a repository in such a way that other researchers are able to replicate your findings and extend them if desired.

We are unable to provide the raw coding data as this data is on paper in notebooks and contains information that identifies instructors. We have supplied in the supporting information the aggregated mean values for each of the PORTAAL practices for each of the daily observations that were recorded on paper. We will also provide the full-data set used for our SEM analysis which includes all 14,000+ student data points for the 46 units of analysis.

We note that the grant information you provided in the 'Funding Information' and 'Financial Disclosure' sections do not match.

We will correct this oversight when we resubmit.

Mary Pat Wenderoth, Teaching Professor
Box 355320 430 Hitchcock Hall Seattle, WA 98195-1800
206.685.8022 mpw@uw.edu https://sites.google.com/site/uwbioedresgroup/

Reviewer #1: I would really love for the analysis and conclusions of this paper to be correct, but as it stands, there is one very fundamental potential flaw in it that must be addressed. It is possible that there is data showing that this potential flaw is not real, but if that is the case, it needs to be in the paper. However, if that is not the case, I believe that the work is fundamentally flawed and, unfortunately, not appropriate for publication. However, if this problem can be addressed by a small amount of additional data and explanation, then I could provide a more detailed and largely favorable review. However, I need to see what they say about this issue before digging deeply into the rest of the analysis for further review, since it is so relevant. So I would hope they might revise and resubmit.

The potential flaw is the implicit assumption of no correlation between the nature of the exams and their grading on one hand, and the views and teaching practices of the instructor on the other. They do structural equation modeling looking at how exam scores across a variety of courses correlate with various teaching practices, with the assumption that these practices result in better exam performance. However, if the exams and grading policies are set by the individual instructors, then an alternative and likely more plausible explanation of their results is simply that teachers who are more inclined to use the various PORTALL practices are also likely to give exams on which students get better grades. In that case, all they are showing is an indirect and not very interesting correlation, teacher attitudes about learning impact both their teaching and exam practices. It says nothing about the impact of teaching practices on learning

My concern is not an abstract speculation. I have done a moderate amount of analysis of university science exams and their grading, and I have seen how wildly variable they are in character and quality. Right now, I am trying to get two courses changed in which the instructors are adamant about neither letting students see the correct answers to the exam questions and/or the basis on which their individual exams were graded. I know of other courses where the exams are largely puzzles covering material related to but not covered in class, and others where the grading makes no logical sense. In these, and other examples I could unfortunately give, the exam score is largely unrelated to mastery of the material, and instead primarily depends on figuring out the idiosyncrasies of the instructor. I have also seen that generally those faculty who are inclined to use more active learning practices are also more inclined to have more meaningful and transparent exams and grading.

In this paper, the only description of the exams is to say they are given by the different instructors, and the researchers rated them according to Bloom's level. It is true that higher Bloom's level questions are generally associated with greater difficulty and lower scores, that is often only a small part of what determines the score on an exam question. The many other instructor idiosyncrasies matter a great deal, including such simple features as whether the instructor believes the average grade should be 50% or 80%, again, a difference I have observed in practice.

Mary Pat Wenderoth, Teaching Professor

Box 355320 430 Hitchcock Hall Seattle, WA 98195-1800

206.685.8022 mpw@uw.edu https://sites.google.com/site/uwbioedresgroup/

What I have discussed are examples of the extreme but actual cases where exam scores are strongly dependent on instructor attitudes, and hence tend to also correlate with their attitudes about teaching practices. That is not always true, however. I have also seen departments where the exams and grading policies were tightly controlled by the department. Instructors were quite constrained as to what questions they gave and how they were graded, in some cases even having exams of the large courses created by a committee separate from the course instructors. If something like that was the case for the courses analyzed in this work, my concerns would vanish.

So I cannot claim that this work is flawed, only that this question of the nature of the exams and grading and how sensitive and variable these are according to individual instructor preferences needs to be addressed carefully. As I said above, I would be happier if they had evidence showing the exams were largely independent of instructor idiosyncrasies, and so the claims of the paper were justified, but until this is shown, the paper is not suitable for publication.

Thank you for helping us better clarify the exam data for our audience. We agree with reviewer #1 that exams are a less than perfect means to assess the learning of students and have many drawbacks which reviewer #1 has identified. To address this issue we have added the following thoughts to the METHODS and LIMITATIONS sections of the paper.

This research is a retrospective study of the teaching methods used by faculty at an R1 in the Northwest. At this university all these classes were videotaped using lecture capture technology installed in the classrooms and these recordings are archived. Faculty involved in the study were not involved in any formal professional development and were not specifically trained in the use of the teaching practices associated with PORTAAL. Some of the faculty had read the PORTAAL paper as part of a faculty learning community where multiple DBER articles were discussed over a two year period. Most of the class videos were of classes taught prior to the publication of the PORTAAL paper. Faculty were merely asked if we could review videos of past classes to determine if a new tool (PORTAAL) we were developing could differentiate between courses using various levels of active learning. As this is a retrospective study, compositions of exams in these courses could not be changed to alter student academic performance.

Ours is a retrospective study as we were able to use archived recordings of class sessions in our analysis. Therefore the faculty wouldn't have been able to change exam questions or exam performance as all this material was also archived. We agree that exams (summative assessments) can be of varying quality and certainly do reflect the idiosyncrasies of the instructor. Though the process of exam writing and grading are often less ideal than we would desire, exam performance is often the main factor that determines if a student proceeds to the next level of their academic career. We also realize that for many courses, other formative assessments or laboratory work may contribute to a student's

Mary Pat Wenderoth, Teaching Professor
Box 355320 430 Hitchcock Hall Seattle, WA 98195-1800
206.685.8022 mpw@uw.edu https://sites.google.com/site/uwbioedresgroup/

grade and we determined that the design and grading of these other assessment forms was too varied for us to use as a proxy for learning.

Reviewer #2: This article looks at the relationship between PORTAAL practices (evidence-based pedagogies) and student exam performance taking into account Bloom's level of exams and several demographic factors of students. It shows a relationship between various PORTAAL practices and performance and some that even differential impact men and women. It is an exciting study that has potential to shape the way in which we, as teachers, design our active learning classrooms. I have a few comments that I think will help to strengthen this paper. I am listing them below in no particular order of importance (just in the order that I encountered them while reading).

1. On page 5, line 127, the authors make the claim that "Bloom level of exams can then be used to control for the cognitive challenge level of exams, as students would be expected to perform better on easier (low Bloom) versus harder (high Bloom) exam questions. I worry a little bit about the way this is stated and it appears the authors are conflating Blooms level with difficulty. I would agree that the lower levels of Blooms have been considered less cognitively challenging and higher levels of Blooms more cognitively challenging (often referred to as LOCS and HOCS; see Crowe, A., Dirks, C., Wenderoth, M. P. (2008). Biology in Bloom: Implementing Bloom's Taxonomy to Enhance Student Learning in Biology. [J] Cbe-Life Sciences Education, 7, 4: 368-381. doi:10.1187/cbe.08-05-0024 and Zoller, U. (1993). Are lecture and learning compatible? Maybe for LOCS: Unlikely for HOCS [J]. Journal of Chemical Education, 70, 3: 195-197. doi: 10.1021/ed070p195). However, cognitive difficult is often mistakenly conflated with performance (see Lemons, P. P., Lemons, J. D. (2013). Questions for Assessing Higher-Order Cognitive Skills: It's Not Just Bloom's. [J] Cbe-Life Sciences Education, 12, 1: 47-58. doi:10.1187/cbe.12-03-0024 and Wyse, A. E., Viger, S. G. (2011). How item writers understand depth of knowledge. [J] Educational Assessment, 16, 4: 185–206. doi: 10.1080/10627197.2011.634286).

Often, performance doesn't reflect the actual cognitive difficulty assigned by Bloom's Taxonomy (see Momsen, J. L., Long, T. M., Wyse, S. A., Ebert-May, D. (2010). Just the Facts? Introductory Undergraduate Biology Courses Focus on Low-Level Cognitive Skills. [J] Cbe-Life Sciences Education, 9, 4: 435-440. doi:10.1187/cbe.10-01-0001), as many other factors may play a role in that difficulty (see Jensen, JL, Phillips, AJ, & Briggs, JC. (2019). Beyond Bloom's: Students' Perception of Bloom's Taxonomy and its Convolution with Cognitive Load. Journal of Psychological Research, 1(1): 1-9.). I might just rephrase this to say that the Blooms level can be a proxy for cognitive challenge but may or may not reflect performance.

On the other hand, an easy way to test this is to do a quick analysis between assigned bloom level (low v high) and performance on a few selected tests to see if you can indeed see a strong and robust relationship between the two. You would probably need to do this individually for each instructor as exam writing

Mary Pat Wenderoth, Teaching Professor

Box 355320 430 Hitchcock Hall Seattle, WA 98195-1800

206.685.8022 mpw@uw.edu https://sites.google.com/site/uwbioedresgroup/

styles and class structures likely play a role in whether or not cognitive level correlates with performance.

Thank you for these comments and for taking the time to suggest all the valuable citations on this topic. We have incorporated the majority of these citations in the appropriate locations in the text.

1. *Our current SEM results show that student exam performance is negatively correlated with Bloom's level, which is in agreement with our earlier work (Freeman, Haak, & Wenderoth, 2011, see Fig. 2).*
2. *We have added the following thoughts to the paper.*

To attempt to control for the varying cognitive challenge of exams across all courses, we categorized each exam question according to Bloom's Taxonomy of Cognitive Domains (Bloom, 1956; Crowe et al., 2008). We realize that all attempts at categorizing exam questions have limitations. The challenge level of the exam can be influenced by many factors including but not limited to the following: the length and format of the question, wording of the question stem and distractors (Kibble, 2017) cognitive load of the question (Phillips, Briggs, & Jensen, 2019) and the alignment of the test with the level of instruction delivered (Lemons & Lemons, 2013; Wyse & Viger, 2011; Wiggins & McTighe, 2005).

Based on our previous research findings (Crowe et al., 2008; Freeman et al., 2011) and others (Stanger-Hall, 2012) we find Bloom's Taxonomy a reasonable proxy for academic challenge level which we realize can differ from performance level. We acknowledge that questions at the lower level of Bloom's taxonomy can be quite difficult for students and lead to lower performance (Momsen et al., 2010) as these questions can address minutia or require knowledge of very specific and often obscure differences between answer choices. However, previous research indicates a negative correlation between Bloom's level and student exam performance (Freeman, Haak, & Wenderoth, 2011, see Fig. 2). Given these findings, it is important to determine how teaching methods impact student exam performance on exams at both low and high Bloom's levels. As most faculty indicate on their syllabi that they want students working at higher cognitive levels (Momsen et al., 2010) in their courses, it is important to determine which teaching practices provide the best type of practice needed for students to excel at these higher levels of cognition.

In any case, it is important to determine how teaching methods impact student exam performance on exams at both HOC and LOC levels whether these exams are easy or not.

2. This is a very minor comment but I noticed it a couple of times in the paper. You are misusing colons. Colons only follow a complete sentence. So, you can say, "...limited to the following:", but not "…limited to:". Alternatively, you can leave the colon out. Colons never come in the middle of a sentence or thought.

Thank you for this comment. We have searched the document for colons and either removed them or corrected the grammatical use of the colon.

Mary Pat Wenderoth, Teaching Professor
Box 355320 430 Hitchcock Hall Seattle, WA 98195-1800
206.685.8022 mpw@uw.edu https://sites.google.com/site/uwbioedresgroup/

3. On page 10, line 239, under Bloom categorization of exam questions, can you clarify how many? and did all researcher rate all problems or did you need to establish IRR and then everyone divided and conquered?

Thank you for this comment. We have added more description of the coding process to the methods section.

“Two trained researchers (authors M.P.W. and M.A.J.) with expertise in biology independently coded all exam questions using the six levels of Bloom’s Taxonomy from a Bloom level 1 (knowledge) to Bloom level 6 (evaluation). The researchers then met to reconcile any differences in their codes and come to one-hundred percent consensus.”

4. I found Table 3 to be just a little confusing being exposed to it prior to your results from the low and high analysis. Perhaps you could add a column heading that said, “First Analysis – all together” and then “Second analysis – divided by Blooms level” or something like that. I just had so many questions until I realized this table applied also to result further down.

We have made the suggested changes to Table 3.

5. Page 16, line 344, you say, “…divide the standard deviation of the exam scores…” Instead, say, "we divided". As it reads now, it sounds more like a list of instructions for the reader.

We have made this change to the text.

6. I would strongly encourage you to comment in the RESULTS section on the negative coefficients. It was very confusing and I didn’t begin to make sense of it until the Discussion. Please mention it in the results.

Thank you for helping us clarify this point. We have added the following text in the RESULTS section under research question 2.

“In units of analysis with lower Bloom’s level exams on average, three PORTAAL practices were significantly positively correlated with student exam scores: small group activities, explaining alternative answers, and total student time. Two PORTAAL practices showed a significant negative correlation with exam scores: random call and students working alone.”

7. On that same note, however, I am not convinced by the explanations in the discussions for why these should be negatively correlated in some instances and positively correlated in others (for some of these PORTAAL practices). Let me give some examples:
a. For example, with random call – it helped in high level but hurt in low level. Your explanation for why it helped in high level seems adequate. But, the reasoning for why it hurt in low level seems to be a reason that would apply to both high and low (anxiety). Why would this differentially impact low-level? Is there some kind of evidence that would suggest that instructors who used low-level exams invoked more anxiety? I’m not sure this is an adequate explanation.

Mary Pat Wenderoth, Teaching Professor

Box 355320 430 Hitchcock Hall Seattle, WA 98195-1800

206.685.8022 mpw@uw.edu https://sites.google.com/site/uwbioedresgroup/

We have expanded our explanation of the seemingly contradictory findings for random call to include the following text.

"However, using random call was significantly negatively correlated with student performance when exams were at lower Bloom's levels. Research has shown that random call can induce some anxiety in students, which may decrease students' sense of safety in the classroom and negatively impact exam performance [59,60]. There are multiple ways to implement random call that may alleviate the anxiety [60] and instructors need to determine the procedure that best matches the characteristics of their student population. As instructors in these courses had a low incidence of random call (mean = 0.38 times per class, SD = 1.55), it is possible that students did not have enough opportunities to become comfortable sharing their answers with the whole class, and instead felt anxious about random call. However, this result could also be due to not enough variation in our sample of courses with low Bloom's exams to detect an accurate effect of random calling on exam scores.

When pooling the data across all units of analysis, random call had a positive correlation with exam performance, which may be explained by a higher incidence of random call (mean = 1.49) and a larger variation (SD = 3.16). The more frequent use of random call across all courses may have contributed to students' comfort level with this PORTAAL practice which may help to reduce their anxiety. This could have allowed students to use more of their working memory to learn course material rather than dealing with feelings of anxiety."

b. The same is true of working alone. I can see how (and I totally buy your explanation) working alone first on high-level items would be a benefit. But, I don't see how working alone first would actually harm someone on a low-level question. You sort of hinted at maybe taking time to work along left less time to answer other questions in class, but I'm not sure that's very convincing. If anything, I would predict no effect. Why would it hurt them?

Thank you for pointing out these contradictory results. We have revised our discussion of working alone to include the following text.

"Research [67] indicates that students adapt their study strategies to meet but not exceed the challenge level of the exam. In other words, exams drive student learning. It is possible that in courses that test at lower Bloom's levels, students may not benefit from reasoning through the problem on their own as the answer is usually a factual piece of information that they either know or they do not know. In this case, taking time to work alone may cause more frustration if students are unable to answer the question on their own. We acknowledge that working alone should benefit students at all Bloom's levels of exams, and further research should investigate if there are components of this practice that could be associated with decreased performance on low Bloom's exams."

8. Page 22, line 477, sentence starting with "We defined and used the code alternative answers each time…" I had to read it like 7 times before I understood what you were saying! It would help if you put the code names in quotes or used

Mary Pat Wenderoth, Teaching Professor

Box 355320 430 Hitchcock Hall Seattle, WA 98195-1800

206.685.8022 mpw@uw.edu https://sites.google.com/site/uwbioedresgroup/

italics or something. It took me forever to figure out that 'alternative answers' was a code name.

Thank you for this suggestion, We have added quotation marks to help delineate our coding terms.

9. Throughout the paper, I'm struggling a little to reconcile the two-level distinction you use in the intro and discussion (high blooms and low blooms) with the three-level distinction you used to code items (low--remember/understand, medium--apply/analyze, and high--evaluate/synthesis). Which was used in the analyses? Can you be a little clearer with this?

Thank you for this comment. We have included the following changes to the text in the methods section describing Bloom's categorization of exam questions.

"Given our sample size, we could not parse and analyze three Bloom's levels of exams. Therefore, we grouped units into either low or high Bloom's level exams in the structural equation models, using the median value of the units (median = 53.8) as the cutoff."

10. Page 23, line 50, you make a comment about eastern and western thought and East Asian cultures. This is a weird addition to the paper considering you did not look at East Asian cultures vs. Westerners. It just seems to come out of the blue. I would just leave it out.

Thank you for this suggestion. We have removed this section from the text.

This is a well-written paper and a fascinating study. I look forward to seeing the edited draft!

Thank you for your very helpful comments and encouragement. We hope you are satisfied with the changes we have made to the text.

Sincerely,

Mary Pat Wenderoth

Mary Pat Wenderoth, Ph.D.
Teaching Professor, Dept. of Biology

Mary Pat Wenderoth, Teaching Professor
Box 355320 430 Hitchcock Hall Seattle, WA 98195-1800
206.685.8022   mpw@uw.edu   https://sites.google.com/site/uwbioedresgroup/
