# Supplementary material for: Evidence-based teaching practices correlate with increased exam performance in biology
Source: PLoS One. 2021 Nov 30;16(11):e0260789. doi: 10.1371/journal.pone.0260789 (PMC8631643; doi:10.1371/journal.pone.0260789)
Supplement: Attachment — Submitted filename: Response to Reviewers.docx [file pone.0260789.s013.docx]

November 15, 2021

**To:** Dr. Paulo F. Carvalho, Academic Editor PLOS ONE

**From:** Dr. Mary Pat Wenderoth

**Concerning:** Manuscript PONE-D-21-24236

Evidence-based teaching practices correlate with increased exam performance in biology

Dear Dr. Paulo F. Carvalho,

Thank you for your comments and offering to make a decision without sending the manuscript out for further review. Please thank the reviewer for their very helpful comment on our manuscript.

We are submitting the three documents you requested.

● This rebuttal letter that responds to each point raised by the academic editor and reviewer(s) uploaded as a separate file labeled 'Response to Reviewers'.

● A marked-up copy of your manuscript that highlights changes made to the original version. This is uploaded as a separate file labeled 'Revised Manuscript with Track Changes'.

● An unmarked version of your revised paper without tracked changes. This is uploaded as a separate file labeled 'Manuscript'.

Below are our responses to each of the editor or reviewers’ comments. Our responses are in blue italicized text immediately following the comment.

**RESPONSES**

**Editor comment:** As you will see from the reviews appended below, Reviewer 1 points out that a critical issue is still present in the revised version of the manuscript: the findings might be due to what I'd call a third variable (e.g., that teachers who are more likely to use active learning practices are also more likely to be more lenient in their grading or create easier exams). Although, from my reading, I believe you were careful to not state causal claims and instead refer to the findings as correlations, I believe this issue should be addressed directly in the text. Thus, please address Reviewer 1's concern by directly stating in the text that there are possible 3rd variable explanations to the results (others are possible as well, such as more active learning being more likely in "easier" courses, or at lower level courses) and the steps you took (or could not take) to address this concern. Your previous edits do not directly address this because they focus on generic limitations of using existing data. Also, please make sure to change any lingering causal language to emphasize that the relations you found, although indicative, should not be interpreted as causal.

**Response:** You will see from our track changes document that we have changed any lingering causal language. We have removed the word “impact” and replaced it with “correlated”. In the first paragraph of our SEM section of the Methods where we discuss variables that could be contributing to the residual variance we have added instructor exam grading methods. We have also directly added “possible 3rd variable explanations” in our limitation section.

Our study is an attempt to do an *in vivo* study of how implementation of multiple PORTAAL practices in actual classroom settings across multiple large courses at an R1 institution would correlate with changes in student exam performance. As with all *in vivo* studies, we are constrained by the actual conditions that exist in the *in vivo* setting. We identified multiple factors that we controlled for in our analysis (incoming GPA of the student, gender, first generation status, minority status, academic challenge level of the exam, etc). What reviewer #1 is asking for, standardized grading across courses, is unreasonable to request in a truly *in vivo* study.

**Reviewer #1 comment:** The authors have not adequately addressed my previous concern. I do not think the evidence they present is adequate to support their conclusion. Their conclusion, which is what makes this paper notable, is that use of more active learning ("portaal practices") leads to improved exam performance. I hope that this is true, but it is at least as likely, if not more so, that what they have actually observed is simply that instructors that use more active learning grade easier.

Their conclusion rests on the assumption that the different instructors have some underlying equivalence in their testing and grading practices, which makes it meaningful to compare results across instructors and attribute differences in exam grades to differences in teaching practices. Given how arbitrary and idiosyncratic exam and grading practices are across faculty at US universities, in the absence of evidence that there is some level of consistency which makes this comparison across instructors meaningful, I do not see how their method and conclusions can be justified.

**Response:** We agree with reviewer #1 that exams and grading can be arbitrary and idiosyncratic across faculty at US universities yet exams are the very methods faculty have chosen to use as one means to assess student understanding of course material. In the department at the university in which this study was conducted, the overwhelming majority of the courses have lab associates that help to standardize exams and grading across quarters and across courses. This is the department’s attempt to provide consistency for the students as they move through the curriculum.

In our 2011 paper, we showed that as more active learning was incorporated into the course the academic challenge level of the exams actually increased, thus the exams got harder not easier. In this paper we attempted to control for the academic challenge level of the exam by categorizing each exam question based on Bloom’s taxonomy of Cognitive Domains and then controlled for Bloom level of exams. We have added to the limitation section of the paper Reviewer #1 concern about grading and we have removed all language that might hint at causality from the manuscript. Furthermore, in the manuscript, we are careful to state that our model predicted the results we are reporting.
